# Construct validity of acute morbidity as a novel outcome for emergency patients

**DOI:** 10.1371/journal.pone.0207906

**Published:** 2019-01-02

**Authors:** Fabrizia Schmid, Alexandra Malinovska, Karin Weigel, Tito Bosia, Christian H. Nickel, Roland Bingisser

**Affiliations:** 1 Emergency Department, University Hospital Basel, Basel, Switzerland; 2 Department of Internal Medicine, University Hospital Basel, Basel, Switzerland; 3 Joint Practice Boettcher and Hoeller, Laufenburg, Germany; Beth Israel Deaconess Medical Center, UNITED STATES

## Abstract

**Objectives:**

Validation of acute morbidity as a novel outcome in emergency medicine.

**Methods:**

Construct validity of acute morbidity was established by comparison to other outcomes using linear and logistic regression models.

**Results:**

Data of 4608 patients were analysed. 1869 patients (40.6%) fulfilled the criteria for acute morbidity. Using multivariate analyses, acute morbidity was associated with outcomes such as hospitalisation (OR: 11, 95%-CI 9–13), mortality (OR 15, 95%-CI 6–49), and ICU admission (OR: 46, 95%-CI 25–96). Reliability of the construct “acute morbidity” was estimated using Cohens Kappa, which was 0.96 for intra-rater and 0.94 for inter-rater reliability.

**Conclusion:**

Reliability of the framework for acute morbidity was high. Construct validity was shown by associations with hospitalisation, mortality, and ICU admission.

## Introduction

Emergency department (ED) visits occurring independently of the acuteness of the health status [1–3] are one of the drivers of crowding [2, 4, 5]. Length of stay is prolonged due to crowding, resulting in adverse outcomes [2, 4]. Emergency physicians (EP) focus on serious, life-threatening illness, and the potential need of invasive procedure or intensive care (ICU) [6, 7]. Outcome research focuses on hospitalisation, length of stay, readmission, morbidity, and mortality [8–12]. Certain outcomes, such as hospitalisation and length of stay, are influenced by non-medical factors [12, 13] others, such as ICU admission, by differences in healthcare systems [10], and mortality—being the hardest of outcomes—is infrequent in ED all-comer studies. Therefore, emergency care research should report additional outcomes, such as the acuteness of health status, or “acute morbidity” [6, 14]. Some tools assessing acute morbidity are available, but have no reported applicability [6, 15]. Acute morbidity can be defined as any potentially life-threatening condition, or any condition requiring early intervention to prevent disability, deterioration, or death [16–19]. While there is an association between acute morbidity and mortality, construct validity and reliability has not yet been formally addressed. We therefore adapted a framework of acute morbidity [18] and assessed its reliability and construct validity, using well-described outcomes as comparators.

## Methods

### Study design and setting

This study was performed at the ED of the University Hospital Basel, Switzerland, a tertiary 700-bed hospital with an annual ED census of over 50‘000. Obstetric and paediatric patients are treated nearby. For seasonal reasons, the study was conducted from October 21^st^ to November 11^th^ 2013 and from February 1^st^ to February 23^rd^ 2015, and was approved by the ethics committee northwest- and central Switzerland (236/13, www.eknz.ch) and Swissethics (BASEC = Business Administration System for Ethics Committees).

### Selection of participants

All adult patients presenting to the ED were screened at presentation and prospectively included, if they consented, were capable to answer questions, and did not withdraw their consent before submission of the manuscript. Informed consent was obtained verbally. The ethics committee waived written consent due to the argument that inclusion could decrease, which would impede validity of an all-comer study. Capacity to consent was determined by a short interview conducted by the study personnel at presentation. Patients had to be able to understand the purpose of the study and to be able to answer questions regarding their complaints.

### Study protocol

The study team consisted of medically trained personnel, instructed to collect online data from the hospital’s electronic health records (EHR), and to interview patients and physicians. Twenty-one days of 3 shifts were covered in each period. Findings were documented in a standardized questionnaire, which is shown in S1 Table. The data were double-checked and digitalized by an external institution (Health Care Research Institute AG, Zürich, Switzerland).

### Data collection

Patients presenting were electronically registered and an EHR was created. Study personnel recorded all data between presentation and discharge/disposition. Patients were initially triaged by a nurse or an EP using the German version of ESI (Emergency Severity Index). [20], and the physician’s first impression of disease severity was recorded on a numeric rating scale from 0–10 (0: not ill, 10: extremely ill). All external examinations were recorded. End of ED workup was defined by discharge, disposition, or death.

Patient characteristics (i.e. age, sex, comorbidities, and ICD-10 diagnoses), dispositions (i.e admissions to wards or ICU, length of stay), follow-up presentations, readmissions, and mortality were taken from the patients’ EHR. One year after presentation, survival was recorded with the help of the EHR, phone-calls (with patients, proxies, or family physicians), or the registry office of Basel.

### Medical chart review methods

Medical chart review methods were based on the twelve criteria of Worster [21], which are based on the eight criteria of Gilbert [22]. Eleven of twelve criteria were fulfilled, namely training of abstractors, selection of case criteria, definition of variables, abstraction of forms, monitoring of performance, mentioning and testing of interrater reliability (IRR), identification of medical records, use of sampling methods, planning of missing data management, and institutional review board approval.

Acute morbidity [19] was defined for non-trauma patients and revised for all-comers [18]. After a predefined chart review of 100 patients, Rohacek’s definition was refined. The chart review was independently performed by two physicians, in case of disagreement, a referee (i.e. senior physician) was included. A data abstraction form was used to document results [18].

The refined framework consists of fourteen rationales for the composite endpoint “acute morbidity”: administration of antibiotics, virostatics, antifungals, immunosuppressives, diuretics, anticoagulants, antihypertensives, and procoagulants; the need for invasive interventions, or prolonged monitoring; new neurological deficits, or seizures; fractures, or self-harm. One of the fourteen rationales was attributed—if several rationales were attributable, the one needing the most resources was chosen according to rules of ICD-10 and Swiss Diagnosis Related Groups [23, 24]. A detailed description of the original [18] and refined framework for the classification of acute morbidity is shown in S2 Table.

### Reliability

For intra-rater reliability, 100 charts were extracted by random sampling. After a month, these charts were independently re-assessed by the original rater. For inter-rater reliability, another 100 charts were extracted for comparison of the two ratings before the referee’s decision.

### Definitions

ESI score was defined as the urgency level a patient has to be treated according to the ESI handbook, German version. [20]

For physician’s disease severity rating (PDSR) the question “how ill does this patient look right now” was asked by the study personnel. The answer was given using a numeric scale from 0 to 10 (0: not ill at all, 10: extremely ill).

Resources were defined as the sum of all external resources used during the ED workup as defined by the ESI.

Emergency department length of stay (ED LOS) was defined as the time in minutes from presentation to discharge, disposition, or death.

Hospitalisation was defined according to Swiss law (LOS ≥24h, including immediate disposition to other hospitals).

In-hospital length of stay (LOS) was defined as the days spent in hospital during index hospitalisation.

ICU-admission was defined as any stay on a medical, surgical, or neurosurgical intensive care unit, or on a stroke or intermediate care unit during index hospitalisation.

In-hospital mortality was defined as death between presentation and discharge.

One-year mortality was defined as death within 365 days after the index presentation to the ED.

Follow-up presentation was defined as visit to the ED or the hospital within 365 days after the index presentation.

Readmission was defined as admission within one year after discharge from the index admission.

Charlson Comorbidity Index (CCI) was calculated for all hospitalised patients. The CCI was calculated using the ICD-10 based Halfon-Version coding system [25, 26]. All ICD-10 coding was performed by a team of professional certified coders of the University Hospital Basel.

### Statistical analysis

Overall sample characteristics were determined using univariate analyses. Unadjusted comparison between patients with acute morbidity and categorical or binary variables was performed using Chi-square test or exact Fisher test; T-test or Kruskall-Wallis test were performed for continuous variables. Results are expressed as mean value with corresponding standard deviation (SD), median with corresponding interquartile range (IQR), or as percentage of the study population.

Univariate and multivariate (adjusted for age and sex) logistic regression models were performed to calculate the association between acute morbidity and the binary outcomes (I) hospitalisation, (II) ICU admission, (III) in-hospital mortality, and (IV) one-year mortality. Results are shown as odds ratio (OR) with 95% confidence interval (95%-CI). For the metric variables (I) ED-LOS, (II) LOS, (III) readmission, and (IV) CCI-Halfon, uni- and multivariable linear regression models were performed. Results are expressed as differences of means with 95%-CI. In order to detect the association between acute morbidity and (I) resources and (II) follow-up presentations, uni-and multivariable Poisson regression models were performed (using a quasi-poisson distribution). Results are expressed as estimates (factor of the mean) with 95%-CI.

The inter-rater and intra-rater reliability of the variable acute morbidity was estimated with weighted Cohens Kappa.

A p-value of <0.05 was considered significant for all tests. All calculations were performed with the statistical softwares SPSS (BM SPSS Statistics 22) and R (Version 3.3.1).

## Results

**Fig 1:**
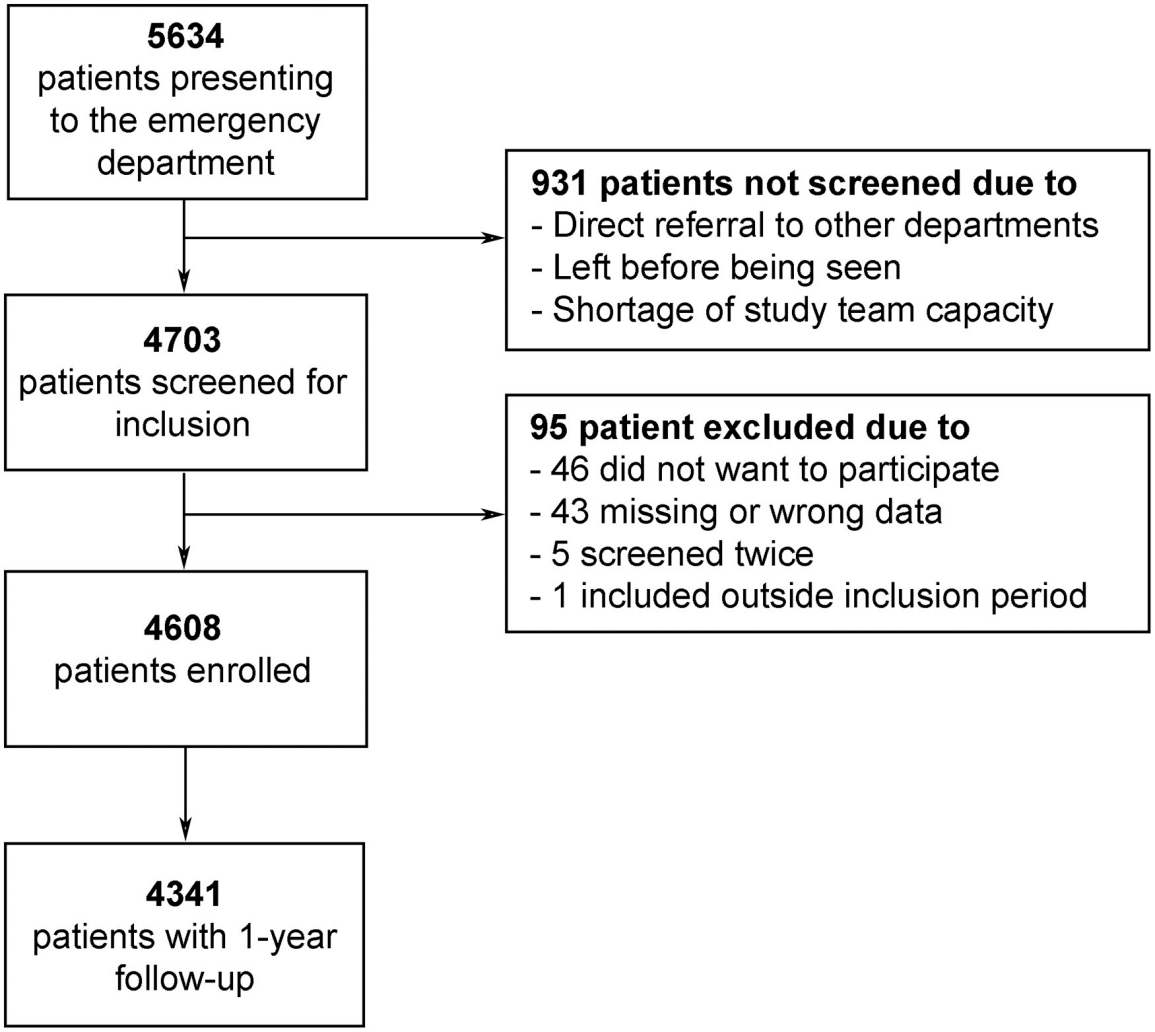
Flow diagram of enrolment.

During the study periods 4703 (83.5%) of all patients presenting to the ED were screened. After exclusion of 95 patients, 4608 patients (81.1%) were included (see Fig 1).

Table 1 shows the patient characteristics and classifications. The median age was 51 years (IQR 33;72). A total of 1869 (40.6%) patients suffered from acute morbidity. Of all patients with acute morbidity, 1203 (64.4%) were hospitalised, 290 (15.5%) were admitted to an ICU, 69 (3.69%) died during hospitalisation, and 208 (11.6%) died within one year after presentation. 267 patients (5.8%) were lost to follow up, 154 (57.7%) being men and 71 (26.6%) being classified with acute morbidity. The median ED LOS for patients with acute morbidity was 236 minutes (IQR 140;362), compared to 160 minutes (IQR 91;264) without acute morbidity. No difference was found for sex. In the group classified without acute morbidity, nine patients were triaged with an ESI of 1, nine patients were admitted to an ICU, and four patients died during hospitalisation. A detailed description of the suspected diagnosis, the discharge diagnosis and the explanatory statement for these cases is given in S3 and S4 Tables.

**Table 1:** Patients’ characteristics.

	All	acute morbidity, yes	acute morbidity, no	p-value	missings
**Population**, n (%)	4608 (100)	1869 (40.6)	2739 (59.4)		
**Age**, median [IQR]	51.0 [33.0;72.0]	64.0 [44.0;79.0]	44.0 [30.0;62.5]	<0.001	
**Male sex, n (%)**	2379 (51.6)	977(52.3)	1402 (51.2)	0.487	
**Physician’s disease severity rating**, median [IQR]	3.0 [2.0;5.0]	5.0 [3.0;7.0]	3.0 [2.0;4.0]	<0.001	68
**ESI**				<0.001	14
**1**, n (%	93 (2.0)	84 (4.5)	9 (0.3)		
**2**, n (%)	1021 (22.2)	617 (33.1)	404 (14.8)		
**3**, n (%)	1783 (38.8)	798 (42.9)	985 (36.1)		
**4**, n (%)	1551 (33.8)	348 (18.7)	1203 (44.0)		
**5**, n (%)	146 (3.2)	15 (0.8)	131 (4.8)		
**ED LOS** (minutes), median [IQR]	190 [107;301]	236 [140;362]	160 [91;264]	<0.001	160
**Hospitalisation**, n (%)	1543 (33.5)	1203 (64.4)	340 (12.4)	<0.001	
**LOS hospital** (days), median [IQR]	5.0 [2.0;10.0]	6.0 [2.0;11.0]	2.0 [2.0;6.0]	<0.001	3065
**ICU**, n (%)	299 (6.5)	290 (15.5)	9 (0.2)	<0.001	
**In-hospital mortality**, n (%)	73 (1.6)	69 (3.7)	4 (0.2)	<0.001	
**One-year mortality**, n (%)	282 (6.5)	208 (11.6)	74 (2.9)	<0.001	267
**Resources**, mean (SD)	2.2 (1.3)	2.6 (1.3)	2.0 (1.2)	<0.001	840
**Follow-up presentations**, mean (SD)	2.1 (3.0)	2.4 (2.9)	2.0 (3.0)	<0.001	
**Readmission**, mean (SD)	0.7 (1.4)	0.7 (1.4)	0.8 (1.3)	0.155	3065

IQR = interquartile range; SD = standard deviation; ESI = emergency severity index; ED = emergency department; LOS = length of stay; ICU = intensive care unit; follow-up presentation = number of follow-up presentations within one year; readmission = number of readmissions within one year

The rationales for acute morbidity are shown in Table 2, the top ten being antibiotics, invasive procedure, fracture, prolonged monitoring, new neurological deficit, immunosuppressives, antihypertensives, anticoagulants, diuretics and seizure. These rationales account for 1806 (96.6%) of all acute morbidity cases. The subgroup “others” includes virostatics, antifungals, pro-coagulants, and risk of self-harm.

**Table 2:** Classifiers of acute morbidity.

	n	%
Antibiotics	522	27.9
Invasive procedure	324	17.3
Fracture	285	15.2
Prolonged monitoring	231	12.4
New neurological deficit	102	5.5
Immunosuppressive drugs	98	5.2
Antihypertensives	79	4.2
Anticoagulants	66	3.5
Diuretics	55	2.9
Seizure*	44	2.4
Others	63	3.4
*Total*	*1869*	*100*

* Non-triggered, start or adaption of anticonvulsive therapy or new diagnosis of epilepsy

The inter-rater reliability was estimated by weighted Cohens Kappa, which was 0.94. Weighted Cohens Kappa for the intra-rater reliability was 0.96.

A significant positive association between acute morbidity and ED LOS, hospitalisation, ICU admission, LOS, in-hospital mortality, one-year mortality, resources and follow-up presentations was found (see Table 3). No significant positive association was found for CCI-Halfon (p = 0.27) and readmissions (p = 0.17). To demonstrate the independent association between acute morbidity and the different variables logistic, linear and Poisson regression models adjusted for age and sex, were used. The results are comparable to the results from the unadjusted analyses (see Table 3).

**Table 3:** Acute morbidity, univariate and multivariate analysis.

	Acute morbidity, univariate analysis			Acute morbidity, multivariate analysis		
	OR	95%-CI	p-value	OR	95%-CI	p-value
Hospitalisation	12.8	11.0–14.8	<0.0001	10.9	9.3–12.8	<0.0001
ICU admission	55.7	30.4–117.1	<0.0001	45.7	24.9–96.4	<0.0001
In-hospital mortality	26.2	10.8–86.2	<0.0001	14.7	6.0–48.5	<0.0001
One-year mortality	4.4	3.3–5.8	<0.0001	2.4	1.8–3.2	<0.0001
	Estimate	95%-CI	p-value	Estimate	95%-CI	p-value
LOS	3.3	2.4–4.2	<0.0001	3.3	2.4–4.1	<0.0001
ED LOS	76.8	66.8–86.8	<0.0001	54.4	44.2–64.6	<0.0001
Readmission	-0.1	-0.3–0.1	0.17	-0.1	-0.3–0.1	0.17
	Exp (Estimate)	95%-CI	p-value	Exp (Estimate)	95%-CI	p-value
Resources	1.8	1.7–2.0	<0.0001	1.5	1.4–1.6	<0.0001
Follow-up presentation	1.2	1.1–1.3	<0.0001	1.1	1.0–1.2	0.01

ICU = intensive care unit; LOS = length of stay; ED LOS = emergency department length of stay; OR = odds ratio; CI = confidence interval; GMR = geometric mean ratio; exp = exponential

## Discussion

The main findings of this study are the excellent reliability of the novel outcome acute morbidity, and a good construct validity. All traditional outcomes (i.e. mortality, ICU-admission, hospitalisation, LOS, etc.) have their disadvantages, such as a low incidence or a high interaction with social and resource-associated factors, taking into account that simple survival is not the ultimate goal for every patient, and most studies in emergency medicine cannot be powered for mortality, as it fortunately is infrequent. Thus, our novel construct “acute morbidity” was associated with other outcomes in order to validate it: First, the association with the outcome hospitalisation is excellent, the age-adjusted OR being 11. As hospitalisation is not only dependent on acuteness of the health status, but also on availability of care (e.g. by proxies or caregivers), other outcomes needed to be addressed. Second, the association between acute morbidity and in-hospital mortality is excellent, the age-adjusted OR being 15. Four patients without acute morbidity died during hospitalisation—all for reasons unrelated to presentation. Further, long-term mortality, though being lightly associated (odds ratio of 2), was much less related with acute morbidity than short-term mortality. This supports the interpretation that acute morbidity and acute mortality are highly associated, not only by coincidence. Third, the association between acute morbidity and intensive care is highly associated—the limiting factor being the interdependence of both outcomes via the rationale “prolonged monitoring”. Therefore, this association must be interpreted with care (see limitations). Nine patients without acute morbidity were admitted to ICU—all nine with conditions unrelated to presentation, e.g. due to postoperative monitoring for non-urgent operations in the course of hospitalisation.

Other outcomes were also highly associated with acute morbidity: hospital length of stay with OR of 3, and ED length of stay with odds of 54. ED-LOS may be a measure of acuteness or severity, as patients with minor illness tend to be discharged early. As of now, all these associations have not been reported in the literature.

Furthermore, patients with acute morbidity had higher use of resources and more follow-up presentations, another possible relation to severity of disease. Of course, even if the associations were statistically adjusted for age and sex, age and co-morbidity must still be taken into account. Patients with acute morbidity were 20 years older (64 vs. 44 y. Interestingly, CCI did not show any difference between morbidity and no acute morbidity (data not shown).

Other studies have brought forward different constructs for acute morbidity, but they focused on acuteness of disease as a predictor rather than an outcome [15, 27]. Relying on disabling disease codes, such measures have advantages predicting e.g. readmission rates with an OR >5, without associations between predictors and outcomes [28]. However, it seems difficult in clinical practice to use such scores on a daily basis as they depend on lists of specific diseases, typically needing information technology (IT) based decision support.

Is the novel outcome of acute morbidity of any practical use? First, there is increasing interest in clinical practice, quality management, and research in outcomes other than survival [6]. Second, this novel outcome is assessable immediately after work-up (after four hours in our population). Therefore, it is the first outcome available in ED patients. According to this definition, the likelihood of further deterioration in patients without acute morbidity seems exceedingly low: Both in-hospital mortality and intensive care occurred in 0.2% of these patients. As one-year mortality is below 3% in these patients, a truly benign outcome may be predicted. It can be argued that identifying 59% of patients without acute morbidity is the focus of emergency medicine anyway—with or without novel constructs. This may indeed be the case, but some arguments merit consideration: First, hospitalisation is one of the most expensive decisions EPs can take. General rules for this decision do not exist, but individual reasoning is most commonly used. Second, teaching junior physicians how to decide—often under pressure—whom to hospitalise, remains a challenge. Constructs, such as acute morbidity, may support rational decisions. Third, further outcome research in emergency medicine is in need of such constructs. Fourth, all rationales of the composite outcome can be operationalised. Therefore, with the aid of computers, all rules can be implemented in EHRs in order to establish the outcome acute morbidity online during ED work-up.

### Limitations

The main limitations are the single centre approach, the lack of an independent validation cohort, and the interdependence between acute morbidity and ICU admission. First, the framework was initially developed and later refined in our hospital with our patients therefore, external validity may be compromised. Additionally, the 6 weeks study period did not cover all seasons, only fall and winter. However, the ED of the University Hospital Basel is comparable to other urban European EDs, as the high number of ongoing multi-centre studies allows for comparisons. Second, an external independent validation cohort could increase external validity. Third, due to one of the predefined rationales (need for prolonged monitoring), the odds ratio for ICU admission is particularly high. We have decided to report these extremely high odds, because only 12.4% of all patients with acute morbidity were attributed to this subgroup of the composite endpoint. Still, a clear bias must be admitted due to this interdependence of the associations between the two outcomes. Generally, in validation of related outcomes, there is often a “separation phenomenon” involved; namely, most events may be associated with a predictor category. E.g. practically no ICU admissions were found in the group without acute morbidity. Therefore, all models tested tend to an infinite OR (high OR with broad confidence intervals). This, however, is a generic problem in related outcomes. Furthermore, 16.5% of all patients presenting were not screened. This was most likely due to crowding, when patients with minor health problems were rapidly discharged or transferred. Hence, these patients could be underrepresented. Yet another bias is due to the missing data for ambulatory patients concerning readmission and CCI. Moreover, 6% of patients were lost to follow up. If a bias could have occurred, it is most likely an overestimation of 1-year mortality, as patients lost seemed less ill (26% acute morbidity vs. 41% acute morbidity in all-comers).

## Conclusion

Acute morbidity is a novel outcome in emergency care research. It was shown to have a good applicability, as reliability is high, and construct validity can be shown by excellent associations with traditional outcomes, such as hospitalisation, mortality, and—with limitations—intensive care. Further studies are needed for external validation.

## Supporting information

S1 Table Study questionnaire. (PDF)

S2 Table Framework of acute morbidity. (PDF)

S3 Table ESI 1 without “acute morbidity”. (PDF)

S4 Table Mortality without definition of “acute morbidity” at presentation. (PDF)
