# Supplementary material for: Construct validity of acute morbidity as a novel outcome for emergency patients
Source: PLoS One. 2019 Jan 2;14(1):e0207906. doi: 10.1371/journal.pone.0207906 (PMC6314600; doi:10.1371/journal.pone.0207906)
Supplement: S1 Table — (PDF) [file pone.0207906.s001.pdf]

Supplemental Digital Content 1: Study questionnaire

„Emerge“ Erhebungsbogen	“Emerge” study questionnaire
**Herkunft des Patienten**	**Origin of the patient**
Mittel-/Nordamerika	Middle-/ North America
Mittelmeerländer	Mediterranean
Südosteuropa	South-eastern Europe
Übriges Osteuropa	Eastern Europe
Türkei	Turkey
Afrika	Africa
Asien	Asia
Nordamerika/Australien	North America / Australia
Mittel-/Südamerika	Middle-/ South America
**Frage an Pflegefachperson Triage:**<br>**Wie krank/verletzt schätzen sie diesen Patienten ein?**	**Question to triage nurse:**<br>**How ill does this patient look right now?**
0-10 (0 = nicht krank/verletzt; 10 = sehr krank/verletzt)<br>Antwort durch: Triagefachperson/Schichtleitung oder andere Pflegekraft	0-10 (0 = not sick/injured at all; 10 = very sick/injured)<br>Answer by: nurse at triage or other nurse
**Frage an den Patienten:**<br>**Wie krank/verletzt fühlen Sie sich gerade jetzt?**	**Question to the patient:**<br>**How ill do you feel right now?**
0-10 (0 = nicht krank/verletzt; 10 = sehr krank/verletzt)<br>Befragung nicht möglich wegen:	0-10 (0 = not sick/injured at all; 10 = very sick/injured)<br>Questioning not possible due to:
**Frage an den Patienten: Wie stark sind die Schmerzen?**	**Question to the patient: How intense is the pain?**
0-10 (0 = keine Schmerzen; 10 = starke Schmerzen)	0-10 (0 = no pain at all; 10 = very intense pain)
**Frage an den Patienten: Warum kommen sie auf die Notfallstation?**	**Question to the patient: Why did you present to the emergency department?**
**Frage an den Patienten: Haben Sie jetzt folgende Beschwerden?**<br>**(Bitte alle Beschwerden markieren die zutreffen)**	**Question to the patient: Do you have any of the following symptoms?**<br>**(multiple answers possible)**
Fieber, Hautausschlag, Kopfschmerz, Schwindel, akute Sehstörung, akute Hörstörung, Nasenlaufen, Schluckschmerzen, Husten, Auswurf, Atemnot, Brustschmerzen, Bauchschmerzen, Übelkeit, Erbrechen, Durchfall, Verstopfung, Beschwerden beim Wasserlassen, Rückenschmerzen, Nackenschmerzen, Armschmerzen, Beinschmerzen, Gelenkschmerzen, Flankenschmerzen, Gelenkschwellung, Beinschwellung, Bewusstseinsverlust, Gefühlsstörung, Lähmungserscheinungen, Gangstörung, Sprachstörung, Müdigkeit, Schwäche, Appetitlosigkeit, Schlafstörung	Fever, rash, headache, dizziness, acute sight disorder, hearing disorder, nasal discharge, dysphagia, cough, expectoration, dyspnea, chest pain, abdominal pain, nausea, vomiting, diarrhea, obstipation, dysuria, back pain, neck pain, arm pain, leg pain, joint pain, flank pain, joint swelling, leg swelling, altered state of mind, numbness, paralysis, gait disorder, speech disorder, fatigue, weakness, loss of appetite, sleep disorder

**Fragen an den Patienten (nur im Falle einer Krankheit)**<br>**Können sie sich vorstellen welche Krankheit ihre Beschwerden jetzt verursacht?**	**Question to the patient (only in case of a presumed disease):**<br>**Can you imagine which condition provokes your disorders?**
Nein	No
Ja, wenn ja welche?	Yes, if yes which condition?
**Fragen an den Patienten:**<br>**Können Sie sich vorstellen nach dem Aufenthalt auf dem Notfall wieder nach Hause zu gehen?**	**Question to the patient:**<br>**Can you imagine to go back home after the work-up at the ED ?**
0-10 (0 = gar nicht; 10 = sehr gut, problemlos)	0-10 (0 = not at all; 10 = absolutely)
**Frage an den Patienten:**<br>**Sind Sie in den letzten 12 Monaten schon einmal in irgendeinem Spital hospitalisiert gewesen?**	**Question to the patient: Have you been hospitalized in the last 12 months?**
Nein	No
Ja	Yes
Einmal oder mehr als einmal?	Once or more than once?
Über den Notfall? ja oder nein	Via the ED? Yes or no
**Fragen an den Angehörigen:**<br>**Wie krank/verletzt schätzen Sie diesen Patienten ein?**	**Question to the proxies: how ill does this patient look right now?**
0-10 (0 = nicht krank/verletzt; 10 = sehr krank/verletzt)	0-10 (0 = not sick/injured at all; 10 = very sick/injured)
Befragung nicht möglich wegen:	Questioning not possible due to:
**Frage an den Arzt:**<br>**Wie krank/verletzt schätzen Sie diesen Patienten ein?**	**Question to the physician:**<br>**How ill does this patient look right now?**
0-10 (0 = nicht krank/verletzt; 10 = sehr krank/verletzt)	0-10 (0 = not sick/injured at all; 10 = very sick/injured)
**Angaben zum Eintrittsmodus (nur eine Angabe möglich)**	**Details about the mode of arrival (only one answer possible)**
Selbstzuweisung (ohne Rettungsdienst oder Arztzuweisung)	Self-referral (without emergency transport, medical referral)
Zuweisung durch Hausarzt/Grundversorger/Primary Care	Referral by general practitioner/primary care
Zuweisung durch einen anderen Arzt/Spezialisten	Referral by another physician/specialist
Zuweisung durch ein anderes Spital/Ambulatorium	Referral by other hospital/ambulatory
Zuweisung durch eine medizinische Fachperson (Ärztefond, Callcenter)	Referral by medical specialist (callcenter)
Zuweisung durch Notfallpraxis	Referral by emergency practitioner
Über Rettungsdienst mit Notarzt	Referral by emergency medical services with emergency physician
Über Rettungsdienst ohne Notarzt	Referral by emergency medical services without emergency physician
Polizei, Feuerwehr, andere	Police, fire department, other
**Eintrittsort (nur eine Angabe möglich)**	**Place of arrival (only one answer possible)**
Notfallstation	Emergency department
Notfallpraxis	Emergency practice
**Eintritt ins Spital (Zeit, Datum)**	**Time of arrival (time, date)**

**Zeitpunkt Triage (Zeit, Datum)**	**Time of triage (time, date)**
**Triagekategorie (Emergency Severity Index)**	**Triage category (Emergency Severity Index)**
1-5	1-5
**Vitalparameter bei der Triage**	**Vital parameters at triage**
Blutdruck (mmHg)	Blood pressure (mmHg)
Puls	Pulse
Atemfrequenz	Respiratory rate
SpO2 (%)	SpO2 (%)
Temperatur (°C)	Temperature (°C)
**Zeitpunkt Erstkontakt Arzt**	**Time of physician**
**Zeitpunkt Analgesie Beginn**	**Time of analgesia**
Oder: Patient wollte keine Analgesie / ist anbehandelt	Or: patient refuses analgesia / treatment started before presentation
**Ist eine Schmerzerfassung vom Behandlungsteam erfolgt?**	**Was the pain intensity documented on arrival?**
Ja	Yes
Nein	No
**Zeitpunkt Kontakt Oberarzt**	**Time of senior physician**
**Wurde ein Kaderarzt/Senior bei der Betreuung hinzugezogen?**	**Was a senior physician directly involved in the treatment?**
Nein	No
Ja: telefonisch oder vor Ort	Yes: by phone or in the ED
**Wie wurde der Patient direkt nach der Verlegung aus der Notfallaufnahme weiter versorgt? (nur eine Antwort möglich)**	**How was the patient treated directly after the transfer from the emergency department? (only one answer possible)**
Patient hat die Notfallaufnahme vor Behandlungsabschluss verlassen	Patient left the ED before completion of the treatment
Ambulante Versorgung	Outpatient treatment
Stationäre Versorgung	Inpatient treatment
Intensiv-Versorgung	Intensive care
Intermediate Care	Intermediate care
Operative Versorgung innert 24h	Surgery within 24 hours
Stationäre Versorgung auf der Notfall-Bettenstation	Inpatient treatment in the ED observation unit
Verlegung in andere Klinik im eigenen Spital	Transfer to another department in the same hospital
Verlegung in anderes Spital	Transfer to another hospital
Patient während der Notfallversorgung verstorben	Patient died in the ED
**Zeitpunkt Fallabschluss und Bestimmung Zielstation**	**Time of completion of the ED work-up and determination of the final station**
**Zeitpunkt Verlegung bzw. Entlassung**	**Time of transfer or discharge, respectively**
**Eingesetzte Untersuchungen (Mehrfachantworten möglich)**	**Tests performed through ED work-up (multiple answers possible)**
Keine	None

Konventionelles Röntgen, Labor, Sonographie, Echokardiographie, Duplexsonographie, Endoskopie, CT, MRI, interventionelles Röntgen, EKG (nur 12-Kanal-Ableitung)	Conventional x-ray, lab test, ultrasound, echocardiography, duplex-ultrasound, endoscopy, CT, MRI, interventional x-ray, ECG (only 12-derivation)
Konsil (Disziplin)	Consultation by specialist
**Zeitpunkt Anmeldung CT**	**Time of registration of CT scan**
**Zeitpunkt Resultat CT**	**Time of result of CT scan**
**Zeitpunkt Anmeldung MRI**	**Time of registration of MRI**
**Zeitpunkt Resultat MRI**	**Time of result of MRI**
**Zeitpunkt Anmeldung Konsil**	**Time of request of consultation by specialist**
**Zeitpunkt Resultat Konsil**	**Time of written result of consultation**
**Hat es im Verlauf der Notfallversorgung Verzögerungen gegeben?**	**Were there delays in the course of the emergency care?**
Nein	No
Ja	Yes
Diagnostik (mehr als 1.5 Stunden)	Diagnostics (longer than 1.5 hours)
Übernahme durch Station	Transfer to ward
Andere	Other
Wurden dadurch anschliessend nicht notwendige Interventionen am Patienten nötig?	Due to this delay: Have other, possibly unnecessary interventions, been performed?
Ja	Yes
Nein	No
**Aufenthalt im Notfall (nur eine Aussgae möglich)**	**Length of ED stay (only one answer possible)**
Weniger als 24h	Shorter than 24 hours
Länger als 24h	Longer than 24 hours
Länger als 48h	Longer than 48 hours

ED = Emergency department
