# Supplementary material for: Construct validity of acute morbidity as a novel outcome for emergency patients
Source: PLoS One. 2019 Jan 2;14(1):e0207906. doi: 10.1371/journal.pone.0207906 (PMC6314600; doi:10.1371/journal.pone.0207906)
Supplement: S2 Table — (PDF) [file pone.0207906.s002.pdf]

Supplemental Digital Content 2: Framework of acute morbidity

Framework Rohacek	Framework Schmid (revised version)
**New medication (given at the ED)**	**New medication (prescribed at the ED)**
Antibiotics<br>Oral, intravenous (not topical)<br>Not Tamiflu	Antibiotics<br>Oral, intravenous
	Virostatic agents<br>Oral, intravenous (not topical)<br>Not Tamiflu
Diuretics	Diuretics
Anticoagulants<br>Vitamin K antagonist (Marcoumar)<br>Rivaroxaban, Dabigatran, Edoxaban, Apixaban<br>Heparin	Anticoagulants<br>Vitamin K antagonist (Marcoumar)<br>Rivaroxaban, Dabigatran, Edoxaban, Apixaban<br>Heparin<br>(No prophylactical)
Antihypertensives, except diuretics (treatment of hypertensive crisis)<br>Beta-Blockers, Alpha-Blockers,<br>ACE-Inhibitors, Calciumantagonists, AT2-Antagonists, Vasodilatators	Antihypertensives (treatment of hypertensive crisis)<br>Beta-Blockers, Alpha-Blockers,<br>ACE-Inhibitors, Calciumantagonists, AT2-Antagonists, Vasodilatators<br>Not Diuretics
	Blood clotting promoting drugs<br>Thrombin, Prothrombin, Factors concentrates,<br>Specific anticoagulant-antagonists
**Invasive therapies (within 48 hours)**	**Invasive procedure (within 48 hours)**
Operation	Operation
Anesthesia at ED: Ketamin + Dormicum, block anaesthesia<br>(Not nerve block for minor wound care of extermities)	Anesthesia at ED: Ketamin + Dormicum, block anaesthesia<br>(Not nerve block for minor wound care of extremities)
	Intubation

	Permanent urinary catheter
Abdominal puncture	Abdominal puncture
	Pleural puncture, pleural drainage
	Therapeutic lumbar puncture
	Pericardial drainage
	Percutaneous transluminal angioplasty (PTA)
Coronary angiography	Coronary angiography
Gastroscopy	Gastroscopy
Colonoscopy	Colonoscopy
Bronchoscopy	Bronchoscopy
Cystoscopy	Cystoscopy
Colposcopy, hysteroscopy	Colposcopy, hysteroscopy
Laryngoscopy	Laryngoscopy
**Prolonged monitoring**	**Prolonged monitoring**
 Acute stroke, ischemia or transient ischemic attack<br>Epidural bleeding, subarachnoidal bleeding, subdural bleeding or intracerebral bleeding	Cerebral disorders:<br>Circulatory disorders such as acute stroke, ischemia or transient ischemic attack<br>hemorrhage such as epidural bleeding, subarachnoidal bleeding, subdural bleeding or intracerebral bleeding
	Commotio cerebri / craniocerebral trauma
 Myocardial infarction, STEMI, NSTEMI, unstable angina pectoris	Cardiac disorders:<br>Myocardial infarction, STEMI, NSTEMI, unstable angina pectoris<br>Left or right heart decompensation<br>Pericarditis, pericardial effusion<br>Cardiac arrhythmia (not atrial fibrillation)
	Aneurysm or dissection
	Peripheral arterial disease (Fontaine III-IV)
Respiratory failure:<br>Respiratory rate >25 or <8 breaths per minute<br>paO_2 <9.3 kPa	Respiratory failure:<br>Respiratory rate >25 or <8 breaths per minute<br>paO_2 <9.3 kPa

SaO_2 <93% under ambient air	SaO_2 <93% under ambient air
Metabolic disorders:<br><br>Acidosis<br><br>Alkalosis<br><br>Ketoacidosis<br><br>Hyperglycemia	Metabolic disorders:<br><br>Acidosis (pH <7.2 and symptomatic)<br><br>Alkalosis (pH >7.5 and symptomatic)<br><br>Ketoacidosis (even if pH >7.2)<br><br>Hyperglycemia (symptomatic or >20mmol/l)<br><br>Hypoglycemia (symptomatic or <2.8 mmol/l)<br><br>Sodium <131mmol/l (except pseudohyponatremia) or >154mmol/l<br><br>Potassium <3.1mmol/l or >5.9mmol/l<br><br>Calcium <2.1mmol/l or >2.65mmol/l<br><br>Cortisol <200ng/l or stimulated cortisol <500ng/l<br><br>Thyrotoxic crisis
Intoxication<br><br>Intervention or stay at ED for monitoring >3 hours	Intoxication<br><br>Intervention or monitoring >3 hours
Haemodynamic instability:<br><br>sBP <90mmHg<br><br>sBP <100mg and volume therapy (NaCl, ringer lactate)<br><br>Administration of 2 red cell concentrates<br><br>Heart rate > sBP	Haemodynamic instability:<br><br>sBP <90mmHg<br><br>sBP <100mg and volume therapy (cristalloids)<br><br>Administration of ≥ 2 red cell concentrates<br><br>Heart rate/sBP ≥1
Gastrointestinal bleeding<br><br>All gastrointestinal bleedings, except bleed from the anal region (haemorrhoids, etc.), that require red cell concentrates	Gastrointestinal bleeding
**Reanimation**	**Reanimation**
**Anaphylaxis (grade II-IV)**	**Anaphylaxis (grade II-IV)**
	**Seizure**<br><br>Non-triggered<br><br>Primary event of epilepsy<br><br>Start or adaptation of anticonvulsive therapy
**Neurological deficit**<br><br>Caused by diseases of the spinal cord	**New neurological deficit**

Motoric, bladder or rectum dysfunction	
**Fracture**	**Fracture**
Except for luxation conservatively treated	
**Suicide danger**	**Endangermet of self and others**

ED = emergency department; ACE = angiotensin converting enzyme; AT2 = angiotensin 2; STEMI = ST-segment elevation myocardial infarction; NSTEMI = non- ST-segment elevation myocardial infarction; paO_2 = partial pressure of oxygen in arterial blood; kPa = kilopascal; SaO_2 = arterial oxygen saturation; sBP = systolic blood pressure; NaCl = sodium chloride
