# Supplementary material for: Construct validity of acute morbidity as a novel outcome for emergency patients
Source: PLoS One. 2019 Jan 2;14(1):e0207906. doi: 10.1371/journal.pone.0207906 (PMC6314600; doi:10.1371/journal.pone.0207906)
Supplement: S3 Table — (PDF) [file pone.0207906.s003.pdf]

Supplemental Digital Content 3: ESI 1 without “acute morbidity”

First suspicion	discharge diagnosis	explanatory statement
Aortic dissection	Dyspnea of unclear etiology	Suspected diagnosis not confirmed. Patient didn't fulfil any of the criteria for acute morbidity
Aortic dissection	Acute lower back pain	Suspected diagnosis not confirmed. Patient didn't fulfil any of the criteria for acute morbidity
Coma	Isolated seizure	Suspected diagnosis not confirmed.
Smoke intoxication	Smoke intoxication	No need of intervention or prolonged monitoring
Poly-trauma	Spinal contusion	Patient didn't fulfil any of the criteria for acute morbidity
Aortic dissection	Chest pain of unclear origin	Suspected diagnosis not confirmed. Patient didn't fulfil any of the criteria for acute morbidity
Poly-trauma	Contusion of the right side of the body	Patient didn't fulfil any of the criteria for acute morbidity
Seizure	Convulsive syncope	Suspected diagnosis not confirmed. Patient didn't fulfil any of the criteria for acute morbidity
Severe rhabdomyolysis caused by fall	Rhabdomyolysis caused by fall	Patient didn't fulfil any of the criteria for acute morbidity

ED = emergency department; ESI = emergency severity index
