# Supplementary material for: Construct validity of acute morbidity as a novel outcome for emergency patients
Source: PLoS One. 2019 Jan 2;14(1):e0207906. doi: 10.1371/journal.pone.0207906 (PMC6314600; doi:10.1371/journal.pone.0207906)
Supplement: S4 Table — (PDF) [file pone.0207906.s004.pdf]

Supplemental Digital Content 4: Mortality without definition of “acute morbidity” at presentation

ED diagnosis	Cause of death	Explanatory statement
Cachexia of unknown etiology	Exitus letalis due to hospital acquired pneumonia	Hospital acquired pneumonia (LOS 10d)
Epistaxis	Exitus letalis due to suicide	Suicide during hospitalisation (LOS 2d)
Influenza A	Exitus letalis due to previously unknown severe aortic stenosis	Aortic stenosis diagnosed during hospitalisation (LOS 7d)
Postural vertigo	Exitus letalis due to aspiration pneumonia	Hospital acquired pneumonia (LOS 16d)

ED = Emergency department; LOS = length of stay
